# Identification of pathogens causing fever in returning travelers using next-generation sequencing

**DOI:** 10.1186/s41182-025-00763-z

**Published:** 2025-06-13

**Authors:** Kenji Gotoh, Nobuyuki Hamada, Takahito Kashiwagi, Koyu Hara, Hiroshi Watanabe

**Affiliations:** 1 Department of Infection Control and Prevention, Kurume University School of Medicine, 67 Asahi-Machi, Kurume, Fukuoka 830-0011 Japan; 2 Department of Pediatrics and Child Health, Kurume University School of Medicine, Kurume, Japan; 3 Center for the Study of Medical Education, Kurume University School of Medicine, Kurume, Japan

**Keywords:** Febrile returning traveler, Coronavirus, Next-generation sequencing

## Abstract

**Introduction:**

In cases of fever following international travel, medical evaluation should consider the prevalence of infectious diseases in the travel destination. However, there are instances where a definitive diagnosis cannot be made. Identifying these unknown pathogens is crucial for managing febrile returning travelers and as a model for the early detection of emerging infectious diseases. The aim of this study was identification of pathogens from febrile cases where fever was the primary symptom and no other specific clinical features were present.

**Methods:**

Between 2008 and 2020, a total of 164 travelers visited Kurume University Hospital due to illness after returning from abroad. However, despite extensive testing, no definitive diagnosis was reached for 18 febrile travelers. Next-generation sequencing (NGS) was performed on eight samples (five whole blood, one serum, one cerebrospinal fluid, and one nasopharyngeal swab) collected from seven returning travelers with undiagnosed fever. Additionally, virus isolation using VeroE6 cells was conducted on two of these samples.

**Results:**

NGS detected human coronavirus OC43 (HCoV-OC43) genes in all eight samples. Of these, six samples contained only HCoV-OC43 genes, one sample contained both HCoV-OC43 and herpes simplex virus type 1 (HSV-1) genes, and one sample contained both HCoV-OC43 and mumps virus genes. Furthermore, conventional RT-PCR confirmed the presence of HCoV-OC43 genetic fragments in two of the eight samples.

**Conclusion:**

Our findings suggest that before the COVID-19 pandemic, common coronaviruses such as HCoV-OC43 were a frequent cause of fever in returning travelers. If tropical infectious diseases, such as dengue fever and malaria, are excluded and the patient’s general condition remains stable, outpatient follow-up is a viable option.

## Background

In recent years, the number of international travelers has increased significantly, leading to a rise in the number of travelers developing illnesses [1, 2]. Furthermore, the distribution of vector-borne diseases, including Japanese encephalitis, has been expanding, likely due to global environmental changes [3, 4]. Another serious concern is the spread of antimicrobial-resistant bacteria and infectious diseases by foreign travelers [5, 6]. Travel to tropical regions and developing countries, in particular, carries a high risk of infectious diseases, and many returning travelers present with fever of unknown origin [7, 8].

Before the COVID-19 pandemic, there was a significant increase in both the number of Japanese travelers going abroad and foreign travelers visiting Japan, which in turn increased the risk of travelers contracting various infectious diseases [9].

Recently, next-generation sequencing (NGS) has been used to analyze febrile returning travelers, leading to the detection of various viruses, such as dengue virus, Epstein–Barr virus, hepatitis A virus, hepatitis B virus, hepatitis C virus, hepatitis E virus, and chikungunya virus [10, 11]. However, the types and the distribution of infectious diseases vary by region [12], and detailed analyses of where and which infections travelers contract are still insufficient. Additionally, many cases of post-travel fever remain undiagnosed. Analyzing these cases in detail could contribute to better infection control guidance and recommendations for vaccinations during pre-travel medical counseling. Furthermore, accumulating such research could aid in the early detection of emerging infectious disease pathogens like COVID-19 and in predicting pathogens that could cause the next pandemic.

In this study, we report our attempt to identify the causative microorganisms through NGS analysis of samples from patients who developed fever after traveling abroad but could not be diagnosed despite extensive pathogen testing.

## Materials and methods

### Ethical approval

All studies described herein were approved by the Human Ethics Review Board of Kurume University (Approval No. 20263).

### Analysis of returning travelers

We retrospectively reviewed the medical records of returning travelers who visited Kurume University Hospital between 2008 and 2020 and classified them based on factors, such as age, sex, nationality, travel history, symptoms, and clinical diagnosis. We extracted cases of febrile patients whose cause of fever could not be identified despite routine medical diagnostic tests, including blood tests, serological tests, antigen tests, and nucleic acid amplification tests. Whole blood, serum, and throat swab samples were collected from all patients, and additional specimens were obtained as needed based on the patients'symptoms. Stool samples were collected from patients presenting with gastrointestinal symptoms such as diarrhea or abdominal pain. Depending on the travel destination and clinical presentation, a selection of relevant diagnostic tests is performed. Table 1 lists all the diagnostic tests that were conducted at our institution.

**Table 1:** Laboratory tests performed on cases of fever after traveling abroad

Rapid test
Malaria Antigen, Malaria Ag. pLDH/HRP2, Premier Medical Corporation, USA
Dengue virus antigen, OnSite Dengue Ag Rapid Test, CTK Biotech Inc, USA
Dengue virus antibody, OnSite Dengue Ag Rapid Test, CTK Biotech Inc, USA
Chikungunya virus antigen, OnSite Dengue Ag Rapid Test, CTK Biotech Inc, USA
Chikungunya virus antibody, OnSite Dengue Ag Rapid Test, CTK Biotech Inc, USA
PCR test
Adeno virus
Chikungunya virus
Dengue virus
Enterovirus
Hepatitis E virus
Malaria
Rhinovirus
West nile virus
Zika virus

### NGS analysis

Samples that were not appropriately managed or had insufficient volume for NGS analysis were excluded. DNase treatment was not performed; therefore, the extracted samples contained not only RNA but also DNA. Additionally, since DNA viruses involved in active infections are generally replicating and producing RNA transcripts, we considered RNA samples appropriate for pathogen detection. Total RNA was extracted from eight unidentified clinical samples using the QIAamp Viral RNA Mini Kit (Qiagen, Hilden, Germany). Double-stranded cDNA was synthesized using the PrimeScript Double-Strand cDNA Synthesis Kit (TAKARA, Tokyo, Japan). A NGS library was prepared using the Nextera XT Library Prep Kit (Illumina, San Diego, CA, USA) according to the manufacturer’s protocol. NGS was performed using the Illumina MiSeq platform. Host reads were removed by mapping the raw reads to the human reference genome GRCh38 (Genome Reference Consortium Human Build 38). Reads that mapped to the human genome were discarded prior to pathogen detection analysis. The sequencing results were then mapped against reference sequences containing various viral genomes using the CLC Genomics Workbench (Qiagen, Hilden, Germany).

### Virus isolation and RNA extraction

Virus isolation was performed on samples from cases in which the cause of fever had not been identified and sufficient sample volume was available (No. 1 and No. 4). The samples were diluted tenfold and inoculated onto VeroE6 cell monolayers, then cultured in Eagle’s minimum essential medium (MEM) supplemented with 2% fetal bovine serum (FBS) and kanamycin (final concentration: 50 µg/mL) for one week. A single blind passage was performed. Cells exhibiting cytopathic effects (CPE) were frozen and thawed as needed. Total RNA was extracted from cell suspensions using TRIzol™ reagent (Thermo Fisher Scientific, Waltham, MA, USA) according to the manufacturer’s protocol and stored at − 80 °C.

### RT-PCR for confirmation

To reconfirm the NGS results and identify viruses in CPE-positive samples (No. 1 and 4), conventional RT-PCR was performed. RNA samples were re-purified using TRIzol™ reagent, and PrimeScript™ RTase (Takara, Japan) and Takara EX Taq (Takara, Japan) were used for reverse transcription and PCR, respectively. PCR was conducted under identical cycling conditions with different primers (Table 2). The cycle conditions were as follows: 45 ℃ for 1 h (RT step) followed by 94 ℃ for 2 min; 25 cycles at 94 ℃ for 20 s, 60 ℃ for 20 s, and 72 ℃ for 20 s (PCR step). RNA samples were stored at − 80 °C. RT-PCR was also performed for sample No. 7–2, for which residual RNA was available. RNA samples corresponding to NGS numbers 2, 3, 5, and 6 were unavailable and therefore not analyzed.

**Table 2:** Primers for RT-PCR of Human coronavirus OC43

No	Amplicon size (bps)	Name of primer	Sequence
1	409	5KYOC43F	GATGAGTCCTTTTACAAGAACATG
2		5KYOC43R	TAATCATCCACATCGGTCCATTTAC
3	206	coronapolOUTF	TTTGAGTGATGATGGGGTTGTGTGTTATAATTCTG
4		coronapolOUTR	CACCATCCATCTTTACAAGCATTGTGTGTTGTGAA
5	84*	coronapolINF	GTGCCTTTCAACAGGTATTATATTATCAAAATAAC
6		coronapolINR	CATTATTTATGTCATGTTCAACCCAACATTTGGAT

^*^GTGCCTTTCAACAGGTATTATATTATCAAAATAACGTTTTTATGTCAGAATCCAAATGTTG

GGTTGAACATGACATAAATAATG (NGS No. 4, No. 4–2/Vero, and NGS No. 1–2/Vero)

## Results

### Characteristics of returning travelers

A total of 164 returning travelers (149 Japanese and 15 foreigners) visited Kurume University Hospital during the study period. Their mean age was 35.7 years (range: 11 months to 79 years), and 79 were male. Diagnoses and travel destinations are shown in Table 3. The most common diseases included 37 cases of animal bites with rabies risk, 33 cases of traveler’s diarrhea, 21 cases of upper respiratory tract infections, 12 cases of dengue fever, and 12 cases of influenza A or B. Of the 12 dengue cases, 11 were from travelers returning from Southeast Asia, and 5 of the 7 malaria cases were diagnosed in travelers returning from Africa. However, 18 febrile returning travelers remained undiagnosed despite undergoing a comprehensive set of diagnostic tests, including rapid tests and PCR for tropical and viral infections, as summarized in Table 1. These cases highlight the diagnostic limitations of conventional testing approaches.

**Table 3:** The diagnoses and destinations of returning travelers

Diagnosis	No. of patients	Southeast Asia	South Asia	East Asia	Middle East	Europe	Africa	NorthernAmerica	Central and South America	Oceania
Animal bite with rabies risk	37	24	4	4	1	2	0	0	2	0
Traveler's diarrhea	33	16	9	3	1	0	3	1	0	0
Upper respiratory tract infection	21	12	5	0	0	0	1	1	1	1
Dengue fever	12	11	1	0	0	0	0	0	0	0
Influenza A or B	12	8	0	1	0	0	2	0	1	0
Malaria	7	0	2	0	0	0	5	0	0	0
Lower respiratory tract infection	7	3	0	1	0	1	2	0	0	0
Amoebiasis	3	3	0	0	0	0	0	0	0	0
Cellulitis	2	1	0	0	0	0	0	0	1	0
Giardiasis	2	1	1	0	0	0	0	0	0	0
Taenia saginata	2	1	0	0	0	1	0	0	0	0
Lyme disease	2	0	0	0	0	2	0	0	0	0
Aseptic meningitis	2	1	0	0	0	0	0	0	1	0
Typhoid fever	1	0	1	0	0	0	0	0	0	0
Biliary tract infection	1	0	0	0	0	0	0	0	1	0
Cutaneous leishmaniasis	1	0	0	0	0	0	1	0	0	0
Rash (unknown origin)	1	0	1	0	0	0	0	0	0	0
Fever (unknown origins)	18	13	0	1	0	1	3	0	0	0
Total	164	94	24	10	2	7	17	2	7	1

### Characteristics of viral analysis cases

Of the 18 undiagnosed cases, 8 samples from 7 cases were analyzed as they had been appropriately stored and had sufficient specimen volume for NGS. These travelers (3 males and 4 females, aged 5–49 years) had returned from Southeast Asia, South Asia, and Central America. Whole blood, serum, cerebrospinal fluid, or nasopharyngeal swabs were used for viral analysis.

### NGS analysis

**Fig. 1:**
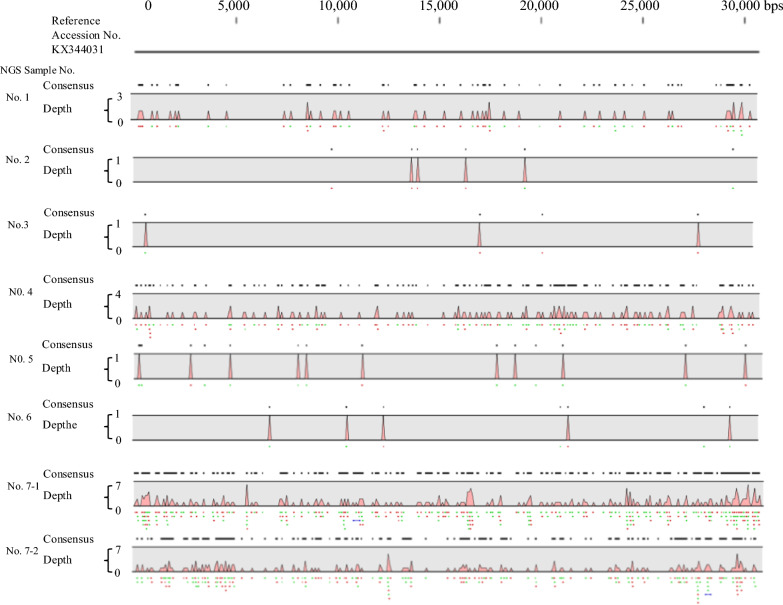
Coverage of next-generation sequencing (NGS) reads from patient samples mapped to the reference genome of the human coronavirus OC43 strain (accession number: KX344031). The upper solid line represents the reference genome. The second, discontinuous line from the top indicates the consensus sequence. Sequence depth across the genome (ranging from 0 to 7 nucleotides) is represented by red (forward strand) and green (reverse strand) dots beneath the red waveform. The red waveform also reflects read depth but is partially omitted for clarity and scale. Depth shows number of reads

NGS detected human coronavirus OC43 (HCoV-OC43) genes in 6 of the 8 samples, HCoV-OC43 and herpes simplex virus type 1 (HSV-1) in 1 sample, and HCoV-OC43 and mumps virus in another sample (Table 4). As shown in Fig. 1, the NGS sequences of patients were identical to the reference genome of the human coronavirus OC43 strain (accession number: KX344031).

**Table 4:** Characteristics of febrile returning travelers and virus analysis

NGS* No	Age	Collection Date (year/month/date)	Sex	Travel history	Symptom	Sample type	Detected gene by NGS*	Confirmed gene by RT-PCR	Virus isolation
1	49	23-03-2016	Male	India	fever	serum	HCoV-OC43 and Herpes simplex virus-1**	HCoV-OC43 (via virus isolation)	HCoV-OC43
2	8	07–08-2013	Female	Indonesia	fever	whole blood	HCoV-OC43	not done	not done
3	19	05–09-2012	Female	Thailand	fever	whole blood	HCoV-OC43	not done	not done
4	26	15-03-2016	Female	Haiti, Jamaica	fever, headache	whole blood	HCoV-OC43	HCoV-OC43 (direct & via virus isolation)	HCoV-OC43
5	46	18-10-2011	Male	Vietnam	fever, headache	whole blood	HCoV-OC43	not done	not done
6	41	01–09-2011	Female	Cambodia	fever	whole blood	HCoV-OC43	not done	not done
7–1	5	30-11-2011	Male	Indonesia	fever, headache	cerebrospinal fluid	HCoV-OC43 and Mumps virus	HCoV-OC43	not done
7–2		30-11-2011				nasopharyngeal swab	HCoV-OC43	HCoV-OC43	not done

^*^next-generation sequencing

^**^CCGCCCGTCTCGTCCAGAAGACCCCGGCGTACCCAGCACC

### Virus isolation

**Fig. 2:**
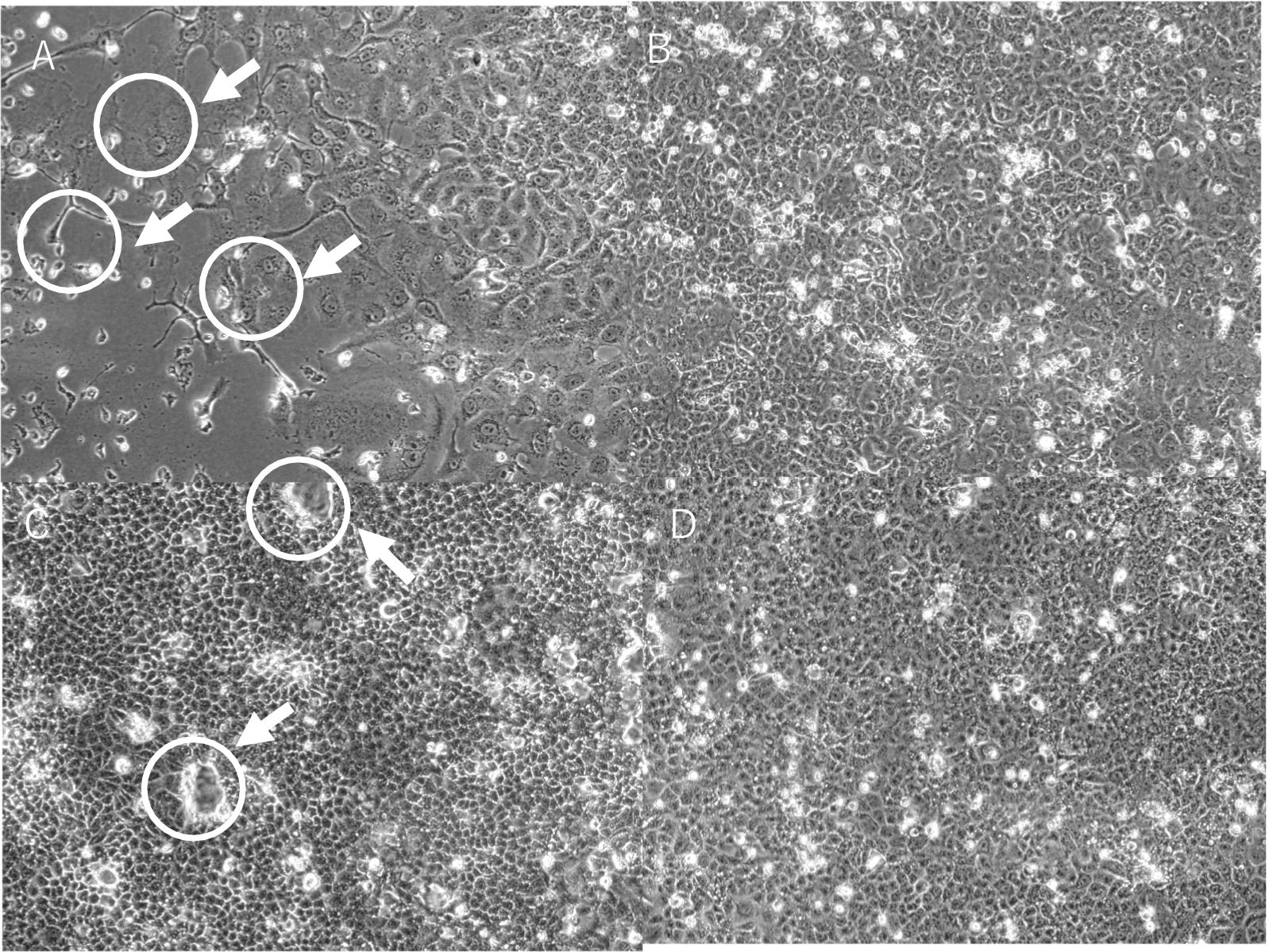
VeroE6 cells inoculated with second-passage NGS samples showing cytopathic effects (CPE). **A**: Cells inoculated with sample from NGS Case No. 1, seven days post-inoculation. **B**: Negative control corresponding to (**A**). (**C**): Cells inoculated with sample from NGS Case No. 4, five days post-inoculation. **D**: Negative control corresponding to (**C**). Syncytium formation, a typical CPE, was observed in (**A**) and (**C**)

Virus isolation was performed using serum from sample NGS* No.1 and whole blood from sample NGS* No.4. As shown in Fig. 2, cells inoculated with samples NGS No.1 and NGS No.4 exhibited syncytia formation after 7 and 5 days, respectively.

### RT-PCR for confirmation

**Fig. 3.**
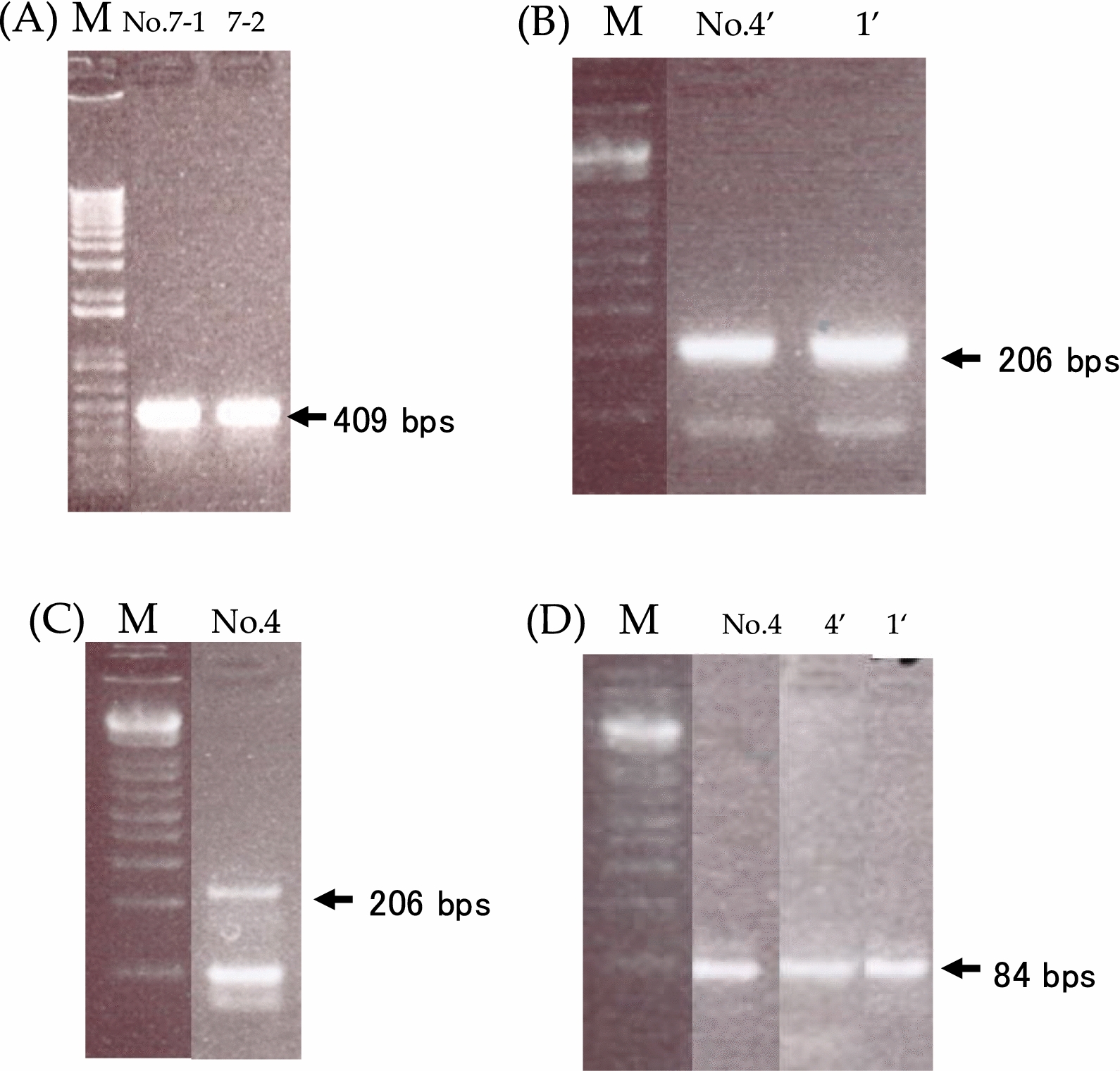
**A** 1% agarose gel electrophoresis of conventional RT-PCR products. The arrow indicates the expected size of the RT-PCR product (409 base pairs). Lane 1: NGS No. 7–1; Lane 2: NGS No. 7–2. The numeric suffix refers to the sample type (see Table 4). (**B**–**D**) 3% agarose gel electrophoresis (NuSieve™ 3:1) of conventional RT-PCR products. **B** Lane 1: NGS No. 4’ from isolated virus; Lane 2: NGS No. 1’ from isolated virus. No bands were observed using 1 µL of each sample. **C** Repeated PCR using the same samples with increased volume. Lane 1: NGS No. 4. The arrow indicates the RT-PCR product size (206 base pairs). **D** Lane 1: NGS No. 4; Lane 2: NGS No. 4 ‘from isolated virus; Lane 3: NGS No. 1’ from isolated virus. The arrow indicates the amplicon size (84 base pairs). “M” indicates the size marker

Based on NGS and virus isolation results, RT-PCR was performed only on NGS*No.1 and No.4, for which virus isolation was successful, and on No.7, for which residual RNA was available (Table 4). PCR was conducted under identical cycling conditions with different primers. The cycle conditions were as follows: 45 ℃ for 1 h (RT step) followed by 94 ℃ for 2 min; 25 cycles at 94 ℃ for 20 s, 60 ℃ for 20 s, and 72 ℃ for 20 s (PCR step). The viruses detected via NGS in samples No.1, No.4 and No.7 were confirmed by RT-PCR (Fig. 3).

## Discussion

As in previous reports, most post-travel medical visits involved cases of animal bites, traveler’s diarrhea, dengue fever, and influenza. In this study, we detected HCoV-OC43 in all tested samples collected from patients with fever of unknown origin. HCoV-OC43 is generally recognized as a cause of respiratory and gastrointestinal infections [13, 14]. Our findings suggest that in cases presenting only with prolonged fever after travel, HCoV-OC43, typically associated with the common cold, may be involved. If tropical diseases, such as malaria, dengue fever, and typhoid fever, can be excluded based on travel history, it may be reasonable to manage such cases as common colds.

Although this study was conducted before the COVID-19 pandemic, our findings suggest that various coronaviruses were already prevalent in Asia.

Our study was conducted at a single center in Kyushu, Japan, which is geographically close to Southeast Asia, and the number of cases was limited; therefore, our findings do not fully reflect the overall situation of febrile illnesses after travel in Japan. In addition, because multiple pathogen genomes were sometimes detected by NGS in a single case, it is not always possible to determine whether each detected pathogen was truly responsible for the fever. Continued epidemiological monitoring of febrile post-travel patients is necessary.

## Conclusion

HCoV-OC43 was detected in all tested cases of prolonged fever in travelers returning to Japan, suggesting it may be a common cause of post-travel febrile illness. This finding highlights its clinical relevance and supports the continued use of NGS for improving diagnosis and management in travel clinics.

## Data Availability

Data is provided within the manuscript or supplementary information files.
